# Effects of noxious stimuli on the electroencephalogram of anaesthetised chickens (*Gallus gallus domesticus*)

**DOI:** 10.1371/journal.pone.0196454

**Published:** 2018-04-26

**Authors:** Amanda E. McIlhone, Ngaio J. Beausoleil, Nikki J. Kells, David J. Mellor, Craig B. Johnson

**Affiliations:** Animal Welfare Science and Bioethics Centre, School of Veterinary Science, Massey University, Palmerston North, New Zealand; University of Bari, ITALY

## Abstract

The reliable assessment and management of avian pain is important in the context of animal welfare. Overtly expressed signs of pain vary substantially between and within species, strains and individuals, limiting the use of behaviour in pain studies. Similarly, physiological indices of pain can also vary and may be confounded by influence from non-painful stimuli. In mammals, changes in the frequency spectrum of the electroencephalogram (EEG) recorded under light anaesthesia (the minimal anaesthesia model; MAM) have been shown to reliably indicate cerebral responses to noxious stimuli in a range of species. The aim of the current study was to determine whether the MAM can be applied to the study of nociception in birds. Ten chickens were lightly anaesthetised with halothane and their EEG recorded using surface electrodes during the application of supramaximal mechanical, thermal and electrical noxious stimuli. Spectral analysis revealed no EEG responses to any of these stimuli. Given that birds possess the neural apparatus to detect and process pain, and that the applied noxious stimuli elicit behavioural signs of pain in conscious chickens, this lack of response probably relates to methodological limitations. Anatomical differences between the avian and mammalian brains, along with a paucity of knowledge regarding specific sites of pain processing in the avian brain, could mean that EEG recorded from the head surface is insensitive to changes in neural activity in the pain processing regions of the avian brain. Future investigations should examine alternative electrode placement sites, based on avian homologues of the mammalian brain regions involved in pain processing.

## Introduction

In the context of animal welfare, pain assessment is important both in terms of identifying when an animal is in pain, as well as in the development of effective analgesic strategies. It is widely accepted that birds, like mammals, are capable of experiencing pain. This belief is supported by multiple lines of evidence. For instance, cutaneous mechanical, thermal, chemical and polymodal nociceptors have been identified in birds, and these respond to noxious stimulation in a similar manner to the equivalent mammalian nociceptors [1, 2]. Further, behavioural and physiological responses to nociceptive stimuli consistent with those observed in mammals have been described in birds subjected to mechanical, thermal and chemical noxious stimulation (e.g. [2, 3, 4]). Opioids and non-steroidal anti-inflammatory drugs that are known to produce effective analgesia in mammals have also been shown to have analgesic properties in birds [5–8]. Finally, like mammals, birds have also been shown to possess endogenous pain modulation mechanisms [9, 10].

It is, therefore, important to have accurate and reliable means for identifying pain in both wild and domesticated birds. In addition to potential pain associated with naturally-occurring disease or injury, commercially-reared poultry may also be prone to acute or chronic pain as a result of management or environmental factors, such as routine beak trimming, feather pecking, footpad dermatitis, bone fractures secondary to osteoporosis, or lameness [11]. To date, there is limited information on pain mitigation strategies in birds. That which is available indicates that birds metabolise analgesic drugs differently to mammals [12], and that the efficacy of specific analgesic drugs can vary considerably between different bird species [13]. As such, there is also a need for reliable means of assessing analgesic efficacy in birds.

Because pain is an inherently subjective experience, its assessment in non-human animals is complicated by the lack of a common language. Therefore, animal pain must be assessed by indirect means. Traditionally, changes in behaviour or physiological indices, such as heart rate, blood pressure and stress hormone levels, have been used to assess pain in animals.

The use of behaviour as an index of avian pain can be problematic, as birds, particularly prey species, may not display overt pain behaviour [14, 15] and when they do, these can vary from fight-or-flight to conservation/withdrawal responses, even within species, breeds, strains or individuals [1, 3, 4]. For example, the behaviour of chickens subjected to repeated feather removal changed from initial agitation and escape behaviour to tonic immobility following successive removals [3]. In other experiments, chickens subjected to electric shock or comb pinch displayed escape/avoidance responses, whilst those subjected to cutaneous thermal or oral nociceptive stimuli displayed passive immobility [1, 4, 16]. As such, there is no single behaviour, or suite of behaviours, that reliably indicate pain in birds. In addition, such variations in the behavioural expression of pain complicate the interpretation of analgesic efficacy in experimental studies, given that behaviour such as immobility may be elicited by pain or by the sedative effects of analgesic drugs [17].

Physiological responses such as changes in heart rate and blood pressure, or changes in hormone secretion such as glucocorticoids or adrenaline, have also been used in avian pain assessment. For example, transient increases in both heart rate and blood pressure were observed in chickens following feather removal; however, there was considerable individual variation in heart rate responses [3]. Despite their relative ease of use, a significant limitation of these measures is that they are not specific to nociception [18] and may also be influenced by non-painful stressors such as exercise, anxiety, handling and restraint [18, 19].

In mammals, quantitative changes in the frequency spectrum of the electroencephalogram (EEG) have been shown to reliably indicate nociception in a range of species [20]. Although still an indirect measure, changes in the EEG frequency spectrum reflect alterations in cortical electrical activity and are therefore more likely to relate to the cognitive perception of pain [21]. Using a minimal anaesthesia model (MAM [20]), in which the EEG is recorded under light anaesthesia using electrodes positioned on the head surface, desynchronisation of the EEG in response to nociceptive stimulation has been reported in horses, sheep, cattle, deer, cats, dogs and pigs [22–26]. The MAM has also been used to assess the efficacy of a variety of analgesic strategies in several species (e.g. [27, 28–30]).

As a research tool, the MAM provides advantages over other pain assessment tools in that it allows cortical responses to noxious stimuli to be studied in the absence of conscious perception, thus permitting the inclusion of a negative control group without compromising the welfare of study animals [24]. In addition, the use of anaesthesia reduces the influence of extraneous variables on the EEG, thereby increasing its specificity.

In birds, EEG recordings have been used extensively to study sleep patterns (e.g. [31, 32–34]) and to assist in determining the level of consciousness during application of stunning or euthanasia methods (e.g. [35, 36–38]). Despite differences in the underlying brain anatomy, the spontaneous EEG recorded from birds shows many similarities with that recorded from mammals [31, 39].

To date, the MAM has not been applied to the study of pain in birds. Anatomical differences between the avian and mammalian brains mean that brain activity contributing to the EEG may arise from different neuronal structures or populations in birds, therefore influencing the ability of the EEG to detect changes in activity relating to the processing of nociceptive information. The aim of the current study was to determine whether noxious mechanical, thermal or electrical stimuli elicited identifiable changes in the chicken EEG, using the MAM.

## Materials and methods

### Animals

Ten 7–8-week-old female Hyline Brown chickens were used. The chickens were sourced from a commercial hatchery and were maintained in groups of 5 on wood shavings substrate under controlled temperature and light conditions (20°C, 12-hour light/dark cycle). Chick Starter Crumbles (Inghams Enterprise NZ, Levin, New Zealand) and water were available *ad libitum*. All procedures were approved by the Massey University Animal Ethics Committee (approval # 08/80, 2008).

### Anaesthesia

Anaesthesia was induced with 0.5–2% halothane vaporised in oxygen (2 L min-1), using either a facemask or an induction chamber. Following loss of righting reflex and muscle tone, the chicken was placed in left lateral recumbency and 0.1–0.2 mL of lignocaine local anaesthetic (Nopaine; Phoenix Farm Distributors Ltd, New Zealand) was applied to the back of the throat to facilitate orotracheal intubation with a 2.5 mm non-cuffed endotracheal tube. Halothane was delivered using a t-piece non-rebreathing anaesthetic circuit and the chicken was ventilated using an intermittent positive pressure ventilation system (Vetronics Small Animal Ventilator VT-5000, Bioanalytical Systems Inc., IN, USA).

An anaesthetic monitor (Hewlett Packard M1025B, Hewlett Packard, Hamburg, Germany) measured and recorded respiration rate and inspired and end-tidal halothane and carbon dioxide (CO_2_) concentrations (vol%). Expired gas was sampled at a rate of 90 mL min-1 from the system end of the endotracheal tube using a low dead-space ET tube connector. Maintenance of body temperature was assisted by placing the chicken on a 37°C water-heated blanket and covering it with a polypropylene blanket to reduce heat loss. Body temperature was monitored using a cloacal thermocouple. Heart rate was monitored via ECG recording (see below). Following instrumentation, end-expiratory halothane was maintained at 0.95 ± 0.1% for the duration of data collection. The anaesthetic monitor was calibrated daily, according to the manufacturers specifications.

### Experimental procedure

Once end-tidal halothane was stable at 0.95 ± 0.1%, fifteen minutes of baseline EEG data were recorded. Each chicken was then sequentially subjected to five noxious stimuli (discrete thermal, mechanical and electrical stimuli and feather plucking from two different regions, as described in Table 1), with a 15-minute inter-stimulus interval. The two feather plucking stimuli were always delivered sequentially, with a shorter 5-minute inter-stimulus interval. With the exception of the two feather plucking stimuli, the order of stimulus delivery was randomised; however, in the first three birds, electrical stimulation was found to elicit leg and body twitching that persisted beyond the stimulus delivery period, affecting subsequent data collection. Therefore, the electrical stimulus was delivered last to the remaining seven birds, with the preceding stimuli delivered in randomised order. Fifteen minutes after delivery of the final stimulus, the chicken was euthanased via an intravenous overdose of sodium pentobarbital (Pentobarb 500, Provet NZ Pty Ltd, Auckland, New Zealand) whilst still under general anaesthesia.

**Table 1:** Description of the five noxious stimuli applied to halothane-anaesthetised chickens during electroencephalogram (EEG) recording.

Stimulus	Description	Duration
Mechanical	Pinching of the skin ventral to the vent, using closed haemostats	5 seconds
Thermal	Application of a 55°C heated metal rod to the skin under the right wing	5 seconds
Feather pluck 1	Removal of one small contour/semi plume feather from the breast, cranial to the keel (F1)	< 1second
Feather pluck 2	Removal of two feathers from the medial distal thigh (F2)	< 1second
Electrical (delivered last)	Application of a 50 V/50 Hz stimulus to the lateral caudal thigh, near the lateral plantar nerve, using two subcutaneous silver/silver chloride wire electrodes positioned 1 cm apart	2 seconds

### EEG and ECG recording

The EEG was recorded using four 27-gauge subcutaneous stainless-steel needle electrodes (Viasys Healthcare, Surrey, England). Following induction of anaesthesia, electrodes were positioned to continuously record EEG from the left and right sides of the brain with inverting electrodes located caudal to the left and right external auditory meatus and non-inverting electrodes located lateral to the comb on the left and right sides of the head (based on the montage for horses described by [40]). The ECG was recorded using stainless steel electrode clips located medio-cranially to the cranial end of the keel bone and 3 cm lateral to the caudal end of the keel bone. A third electrode located lateral to the pelvis served as a common earth for both ECG and EEG recording.

The EEG and ECG electrodes were fed via break-out boxes to separate physiological signal amplifiers (Iso-Dam isolated physiological signal amplifier, World Precision Instruments, FL, USA). The signals were amplified with a gain of 1000 and band-pass 0.1 to 100 Hz. The amplified signals were digitised at a rate of 1.0 kHz and recorded on a Powerlab data acquisition system (ADInstruments Ltd, Australia). The digitised signals were stored on an Apple personal computer for later analysis.

### EEG and ECG analysis

**Fig 1:**
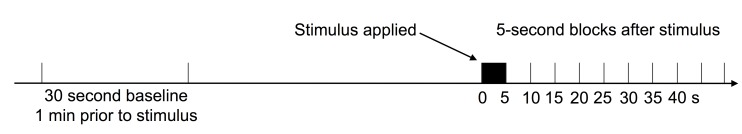
Diagram illustrating the generation of summary data used for statistical analyses of spectral EEG variables.

Raw EEG were manually inspected and any segments contaminated by movement artefact (due to reflex limb withdrawal) were excluded from subsequent analysis. Frequency spectra were subsequently generated for sequential non-overlapping 1 second epochs. Data were low-pass filtered at 30 Hz and Fast Fourier Transformation applied to each epoch (Spectral Analyser, CB Johnson, 2002), yielding sequential frequency spectra with a resolution of 1 Hz. From these, the summary variables total power (P_TOT_; the total area under the frequency spectrum curve), median frequency (F50; the frequency below which 50% of the total power lies) and 95% spectral edge frequency (F95; frequency below which 95% of the total power lies) were derived. EEG data were band-pass filtered between 0.1 and 100 Hz during recording and a 30 Hz high-pass filter was applied prior to Fourier transformation, thus baseline noise was excluded from analyses. Mean F50, F95 and P_TOT_ were calculated for a 30-second block of baseline EEG recorded 1 minute prior to stimulus application and for consecutive 5-second blocks from the time of stimulus application up to 40 seconds post-stimulus. Thus, a total of nine data points were generated per stimulus in each chicken; one before (baseline) and eight after stimulus application (Fig 1). Summary EEG data are provided in S1 Appendix.

Heart rate was determined from ECG data, using the rate meter function in Chart. Mean heart rate was calculated for a 10-second block of baseline ECG recorded 1 minute prior to stimulus application and for consecutive 10-second blocks up to 40 seconds after stimulus application, yielding a total of five data points per stimulus per chicken (one before (baseline) and four after stimulus application). Summary heart rate data are provided in S2 Appendix.

### Statistical analyses

For the purposes of statistical analyses, EEG and heart rate data from each chicken were standardised to a percentage of baseline mean, thus accounting for individual variations in baseline values between chickens.

Data were tested for normality and found to meet the assumptions for parametric analyses. Linear mixed models with autoregressive correlation structure were used to test the effects of stimulus, time, and their interaction, on heart rate and spectral EEG variables. The models included stimulus as a fixed effect, chicken as a random effect and time as a repeated measure. Where significant main or interaction effects were identified, post-hoc pairwise comparisons were performed with Bonferroni correction for multiple comparisons. Post-stimulus values were compared to baseline within-stimulus and values for equivalent time points were compared between stimuli.

All analyses were conducted using SAS Version 9.2 (SAS Institute Inc., NC, USA). Differences were considered significant at P < 0.05. Data are presented as mean ± SEM.

## Results

### EEG analysis

Following application of the electrical stimulus, reflex limb movements occurred in all birds and intermittent limb twitching was common throughout the 40-second post-stimulus period. Following the exclusion of movement-contaminated epochs, no data were available for analysis from the 5-second period immediately following electrical stimulation (0–5). In two birds, persistent limb twitching resulted in the exclusion of data from the entire 40-second post-stimulus period.

**Fig 2:**
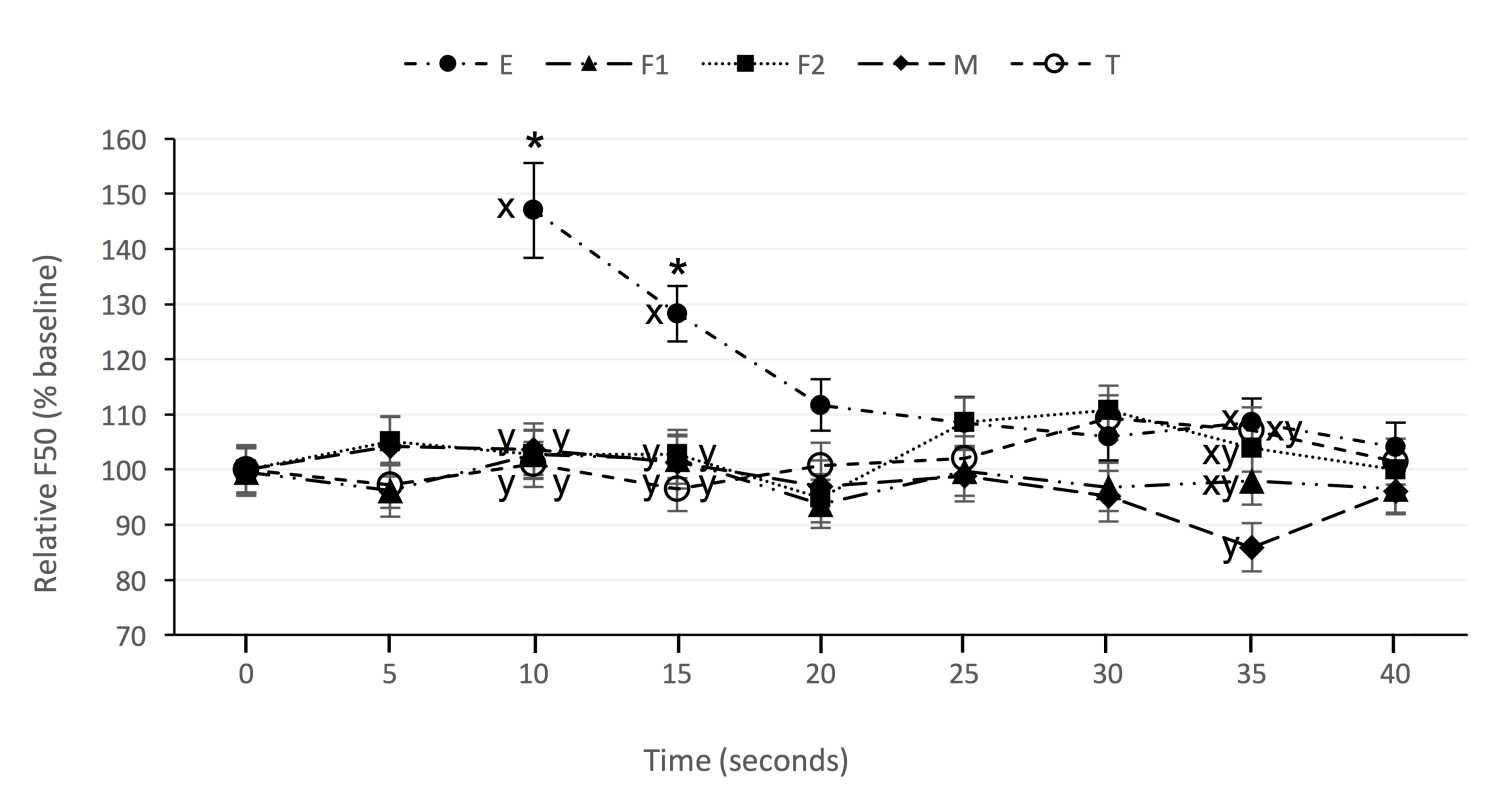
Change in mean (±SE) median frequency (F50) of the chicken EEG at consecutive 5-second intervals after application of noxious electrical (E, closed circle), feather pluck (first feather F1, triangle; second feather F2, square), mechanical (M, diamond), or thermal (T, open circle) stimuli. Asterisks indicate that mean differed to baseline within stimulus (*P* < 0.05). Superscript letters indicate means differed significantly between stimulus type at a common time point (*P* <0.05).

Analysis of variance showed there was no effect of stimulus or time, or their interaction, on the F95 or P_TOT_ of the chicken EEG (Table 2). In contrast, there was a significant stimulus by time interaction effect on the F50. Post-hoc tests revealed that F50 was elevated, relative to baseline, 10 and 15 seconds after application of the electrical stimulus (Fig 2), whereas none of the other stimuli evoked a significant change from baseline. Mean F50 was higher 10 and 15 seconds after application of the electrical stimulus than at the same time after application of a thermal, mechanical, or feather-plucking stimuli (Fig 2). Thirty-five seconds post-stimulus, mean F50 was lower in mechanically stimulated birds than in electrically stimulated birds.

**Table 2:** Effects of stimulus, time and their interaction on the median frequency (F50), 95% spectral edge frequency (F95) and total power (P_TOT_) of the chicken (*n* = 10) electroencephalogram.

	F50		F95		P_TOT_	
	F	*p*	F	*p*	F	*p*
Stimulus	10.27	<0.001	2.44	0.069	1.03	0.407
Time	2.98	0.006	1.37	0.224	1.15	0.312
Stimulus x Time	1.8	0.009	0.88	0.646	0.87	0.674

### Heart rate analysis

**Fig 3:**
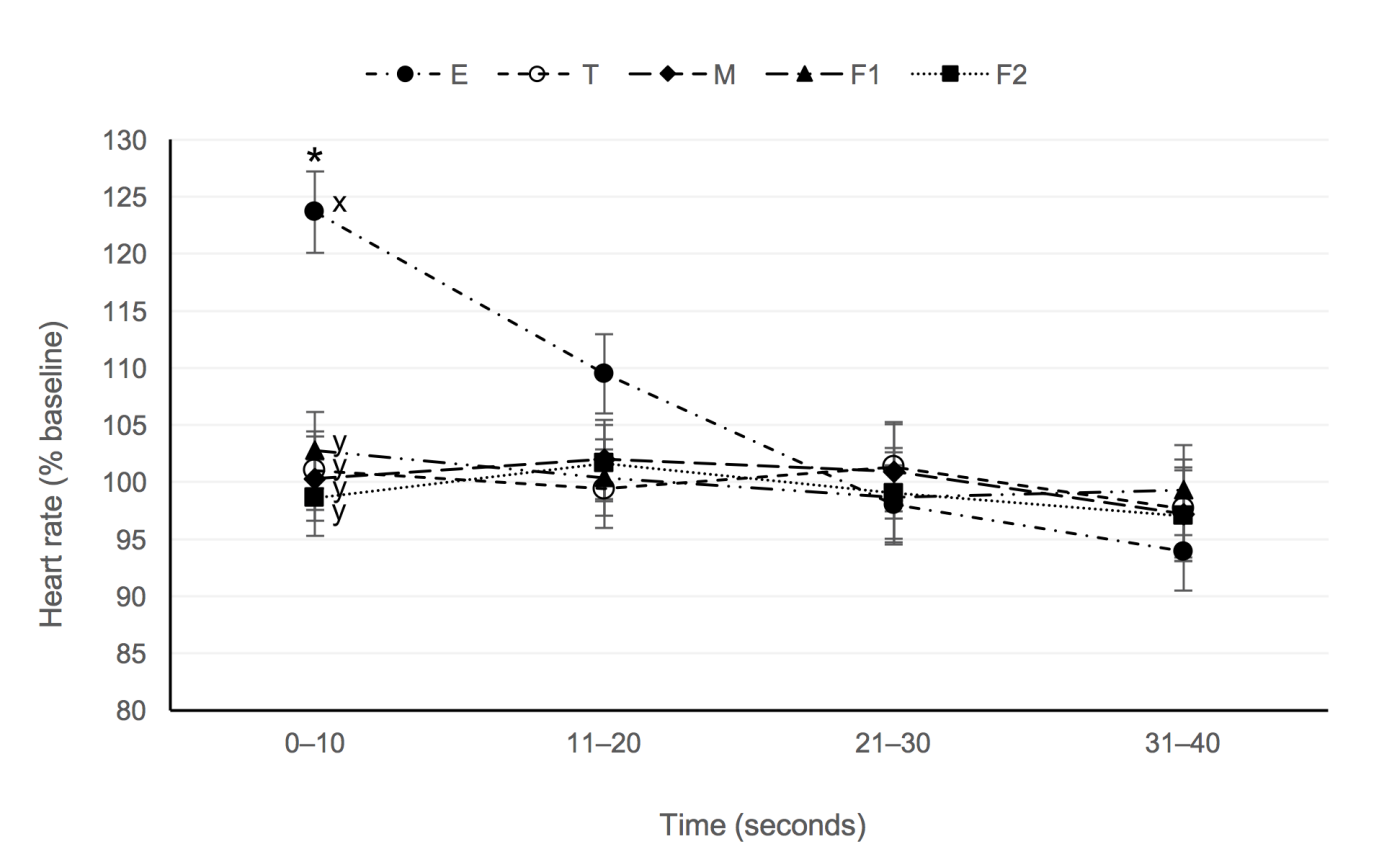
Mean (± SE) heart rate (as a percentage of baseline) following application of noxious electrical (E; circle), feather pluck (first feather F1, triangle; second feather F2, square), mechanical (M, diamond), or thermal (T, open circle) stimuli. Asterisks indicate mean differed to baseline within stimulus (*P* < 0.05). Superscript letters indicate means differed significantly between stimulus type at a common time point (*P* <0.05).

There was a significant stimulus by time interaction effect on heart rate (F = 4.28, P <0.001). Following electrical stimulation, heart rate was greater than baseline in the period 0–10 seconds after stimulus application (P <0.001; Fig 3), but did not differ to baseline in the following periods (P >0.05). No other stimuli elicited a significant change in heart rate. Baseline heart rate did not differ between stimuli (P >0.05).

## Discussion

The aim of this study was to determine whether noxious stimuli elicit quantifiable changes in the EEG of the chicken, using an adaptation of a MAM developed to study nociception in mammals. Noxious stimuli reliably elicit desynchronisation of the mammalian EEG, characterised by an increase in F50 and corresponding decrease in P_TOT_ [20]. Further, prior administration of analgesic drugs abolishes or attenuates EEG nociceptive responses (e.g. [24, 29, 41]), making the MAM a valuable tool for assessing of pain and evaluating pain mitigation strategies in mammals.

This study represented the first attempt to apply the MAM to nociceptive assessment in birds. The application of supramaximal cutaneous thermal or mechanical (skin clamp or feather pluck) noxious stimuli to the chicken failed to elicit changes in any spectral EEG parameter, whilst the application of a noxious electrical stimulus induced a significant increase in F50, but no change in P_TOT_ or F95. Previously, noxious electrical stimulation has been associated with both an increase in F50 and concurrent reduction in P_TOT_ in rats [42] and dogs [28, 43]. As such, the rise in F50 in the current study should be interpreted with caution. Given that electrical stimuli elicit non-selective activation of all local receptors, both nociceptive and non-nociceptive, it has been suggested that non-nociceptive afferent inputs reaching the cerebral cortex might enhance the F50 response to noxious electrical stimulation [42]. It is possible that this might account for the rise in F50 observed in the current study, rather than this being a nociceptive response, given the lack of a corresponding change in P_TOT_.

The possibility that halothane anaesthesia blunted EEG responses to nociception in the present study cannot be discounted. However, this is unlikely, based on recent evidence indicating that halothane induces minimal EEG suppression in the chicken at concentrations of 1.5 MAC or lower [44]. In contrast, isoflurane and sevoflurane cause marked EEG suppression at 1–1.5 MAC and complete suppression at 2.0 MAC [39]. This is consistent with the effects of inhalant anaesthetics on the mammalian EEG, where halothane causes less cortical depression than isoflurane, sevoflurane or methoxyflurane, making it the agent of choice for the MAM [20].

Studies in conscious birds have reported changes in behaviour, heart rate, blood pressure or respiration rate in response to the application of a 50°C thermal stimulus [4, 45], comb pinch [4], feather removal [3] or electrical stimulus [15], suggesting these stimuli are painful to birds. In light of this, it seems unlikely that the lack of EEG changes following stimulus application in the present study indicates the absence of nociception. Instead, it may be that the method employed was unable to detect changes related to nociceptive processing in the avian brain, possibly due to differences in the underlying neural structure.

The avian brain differs to the mammalian brain both in anatomical structure and cellular organisation, particularly in the forebrain region. Whilst both the mammalian and avian telencephalon consist of pallial and subpallial regions, only the mammalian pallium has a laminated neocortex. Mammalian cortical neurons are arranged in complex horizontal layers, with vertically oriented dendritic columns [46]. In contrast, the avian telencephalon has no laminated cortex, instead consisting of an enlarged pallium [47] with a nucleated rather than laminated architecture and radial dendritic projections [46]. As a result, changes in electrical activity in response to discrete stimuli may be more diffuse in comparison to the more localised changes arising from the vertical columnar arrangement of pyramidal cells in the mammalian cortex [48].

Despite differences in the neural architecture, similarities in the patterns of afferent connectivity and distribution of neurotransmitters lead to the hypothesis that distinct nuclei in the avian pallium are functionally homologous to different layers of the mammalian cortex (reviewed by [49]). More recent experimental evidence provides support for homologies between the mammalian cortex and the avian hyperpallium, nidopallium and mesopallium [46–48, 50, 51]. Relative to the mammalian neocortex, these avian pallial structures are less superficial, extending deeper into the brain [52, 53].

Although influenced by activity in deeper regions, the EEG (recorded from the scalp surface) primarily reflects the activity of neurones located in the cerebral cortex [20], which is the most superficial region of the mammalian forebrain. Several widely-distributed regions in the cerebral cortex are known to be involved in pain processing [54, 55]. Among these, the somatosensory, insular, and anterior cingulate cortices are consistently activated in response to noxious stimuli [55, 56], therefore activity in these regions is likely to contribute substantially to the EEG following noxious stimulation in mammals. Whilst the precise location of pain pathways in the avian brain are not known, it is likely that functional homologues in the pallial region are involved. Given the less superficial location of these structures, EEG recorded from the head surface may be insensitive to changes in neuronal activity in these regions.

Similar to the observed EEG responses, a transient increase in heart rate was seen following electrical stimulation only. This contrasts with nociceptive studies in non-anaesthetised chickens, where variable but significant increases in mean heart rate following a comb pinch, 50°C thermal stimulus [4], or feather removal [3] have been reported. It is possible that general anaesthesia may have influenced cardiovascular responses in the present study, given that halothane has been shown to cause cardiac depression in birds [57–59]. Conversely, the increase in heart rate reported in awake birds may have been influenced by non-painful stressors, such as handling or human interaction [19].

## Conclusions

Using the MAM, no consistent evidence of nociception was identified in the chicken EEG following the application of thermal, electrical or mechanical noxious stimuli. It is likely that stimulation of peripheral nociceptors induces quantifiable changes in neural activity in brain areas involved in pain processing in birds and that the inability to detect such changes in the present study relates to the site of electrode placement. Given that proposed avian homologues of the cerebral cortex are located less superficially, electrical activity arising from these regions may not be detectable using electrodes at the scalp surface. Further, the radial dendritic arrangement of neurons in these regions may mean that changes in electrical activity in response to a discrete stimulus cannot be detected at a distance. Future studies should therefore examine alternative electrode placement sites within the avian brain, to determine whether a modified MAM can be applied to the study of nociception and pain mitigation in birds. Whilst the precise location for optimal electrode placement is not known, sites in the avian pallial region believed to be homologous with the mammalian neocortex should be investigated.

## Supporting information

S1 Appendix EEG summary data subjected to statistical analyses. (XLSX)

S2 Appendix Heart rate summary data subjected to statistical analyses. (XLSX)
